# Supplementary figures and images for: Multiple pals gene modules control a balance between immunity and development in Caenorhabditis elegans
Source: PLoS Pathog. 2023 Jul 18;19(7):e1011120. doi: 10.1371/journal.ppat.1011120 (PMC10353827; doi:10.1371/journal.ppat.1011120)

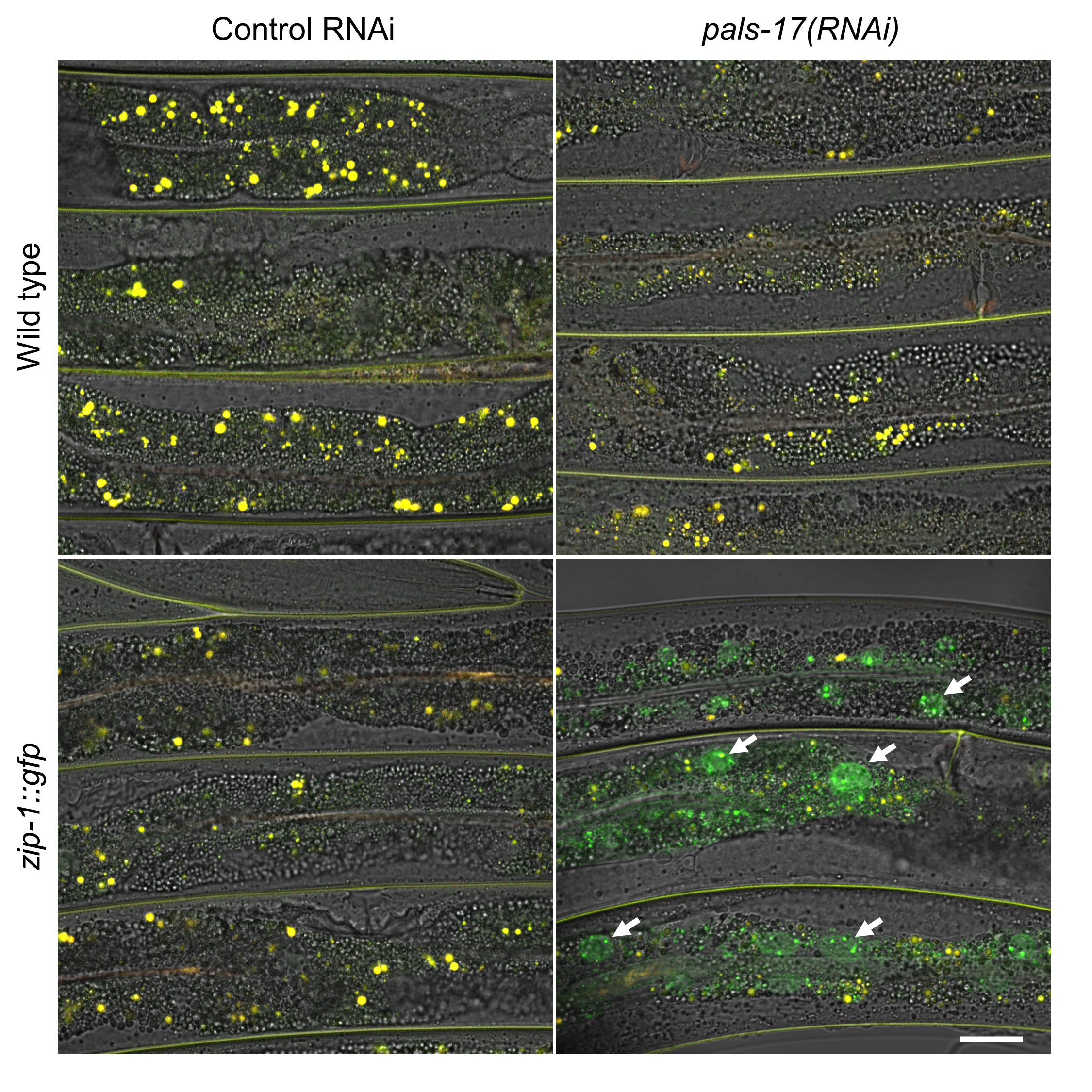

Supplement: S1 Fig — Wild-type and zip-1::gfp animals treated with control and pals-17 RNAi. Green, autofluorescence and DIC channels were merged. Intestinal ZIP-1::GFP expression is indicated with white arrows; autofluorescence from the gut granules and from the cuticle are shown in yellow. Scale bar, 20 μm. (TIF) [file ppat.1011120.s001.tif]

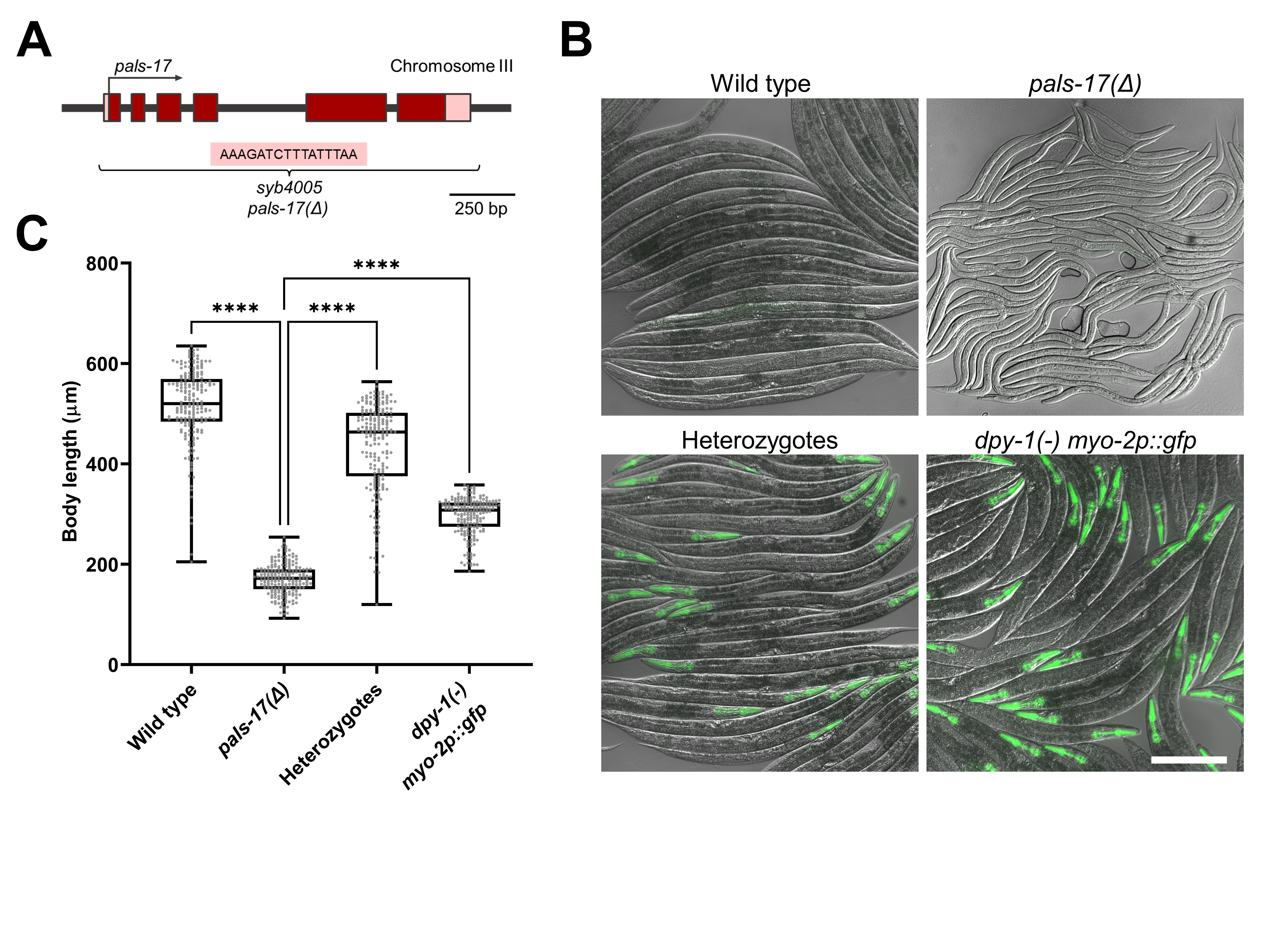

Supplement: S2 Fig — (A) pals-17 gene structure. Exons are indicated with dark red boxes, 5’ and 3’ UTRs shown with light red boxes. The deletion in syb4005 allele (pals-17(Δ)) is indicated by the bracket; the inserted sequence is shown in the pink rectangle. The horizontal arrow indicates the direction of transcription. (B) Synchronized pals-17(Δ) mutants and control strains following 44 h incubation at 20°C. Green and DIC channels were merged. myo-2p::GFP present in the balancer strain is shown in green. Heterozygotes are pals-17(Δ)/sC1(s2023). Scale bar, 200 μm. (C) Box-and-whisker plot of body length values for indicated worm strains. Box lines represent median values, box bounds indicate 25th and 75th percentiles, and whiskers extend to the minimum and maximum values. Gray dots represent individual values for each animal; 50 animals per each of the three replicates were analyzed. A Kruskal-Wallis test was used to calculate p-values; **** p < 0.0001. (TIF) [file ppat.1011120.s002.tif]

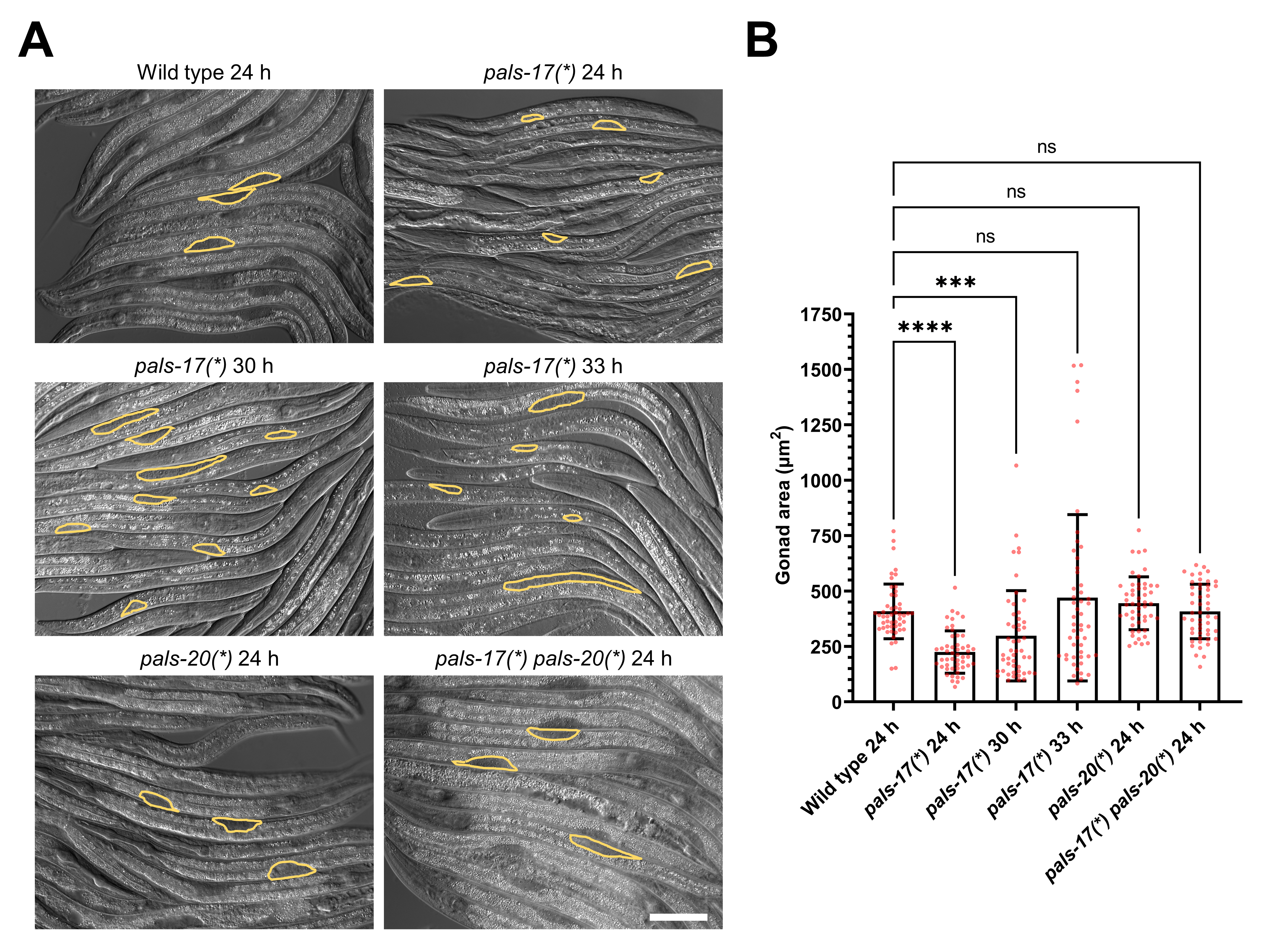

Supplement: S3 Fig — (A) Representative DIC images of wild-type animals, pals-17(*) mutants, pals-20(*) mutants or pals-17(*) pals-20(*) mutants incubated for 24 h at 20°C, and images of pals-17 mutants incubated for 30 h or 33 h at 20°C from L1 stage. The gonads of some animals are outlined with yellow lines for reference. Scale bar, 60 μm. (B) Gonad area measurements. Results shown are the average of two independent experimental replicates, with 25 animals assayed per replicate. Error bars are SD. A Kruskal-Wallis test was used to calculate p-values; *** p < 0.001; **** p < 0.0001; ns indicates no significant difference. (TIF) [file ppat.1011120.s003.tif]

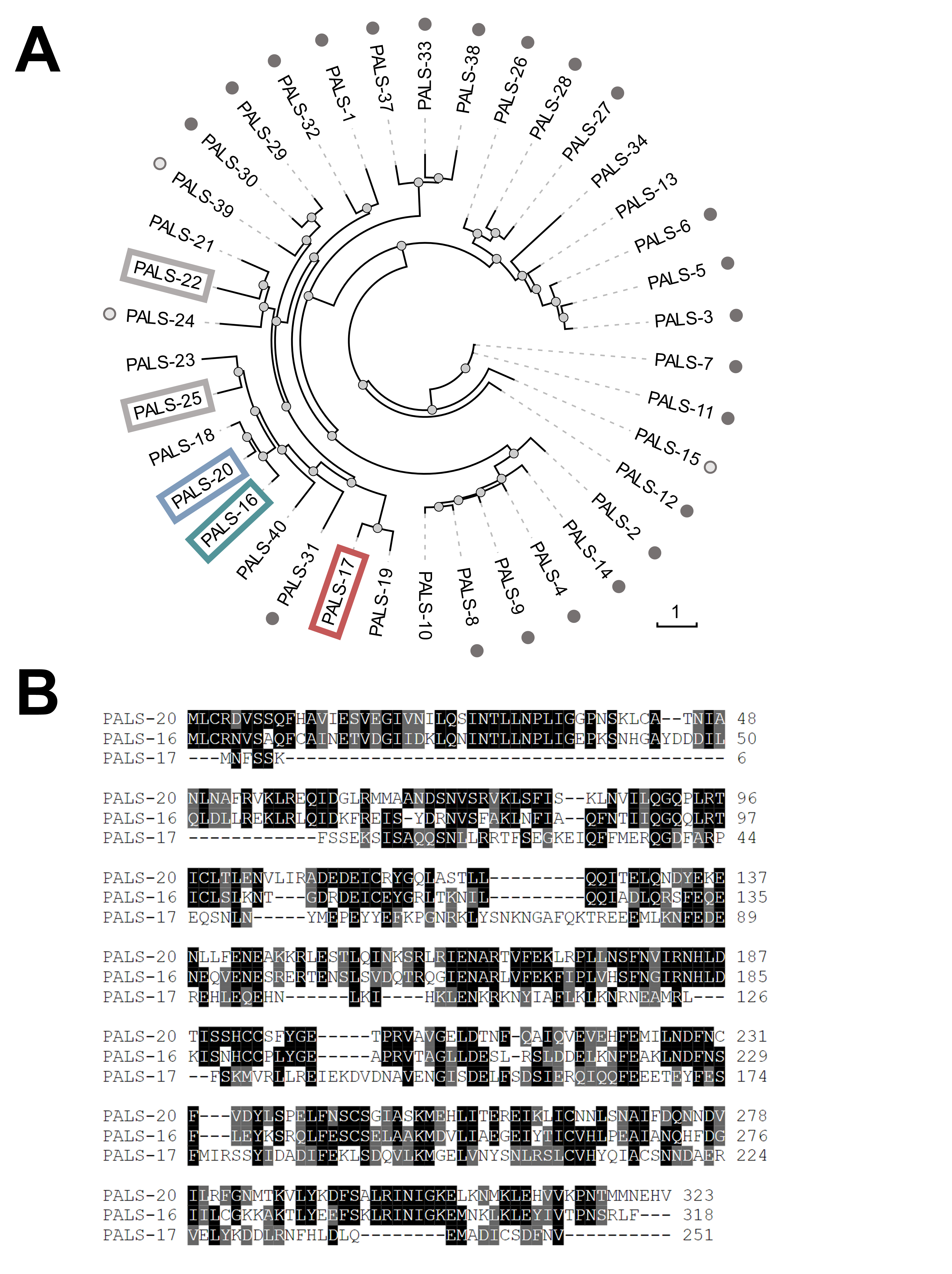

Supplement: S4 Fig — (A) A radial phylogram of the PALS protein family. Dark gray circles next to protein names indicate PALS proteins whose corresponding mRNA levels are significantly upregulated following IPR activation during microsporidia infection and in pals-22 mutants [9,12]. Light grey circles next to protein names label PALS proteins whose corresponding mRNA levels are significantly upregulated only in pals-22 mutant background. The branch length is indicated by the scale bar. (B) Amino acid sequence alignment between PALS-20, PALS-16 and PALS-17. Black boxes indicate identical amino acids; grey boxes indicate similar residues (defined in Materials and Methods). (TIF) [file ppat.1011120.s004.tif]

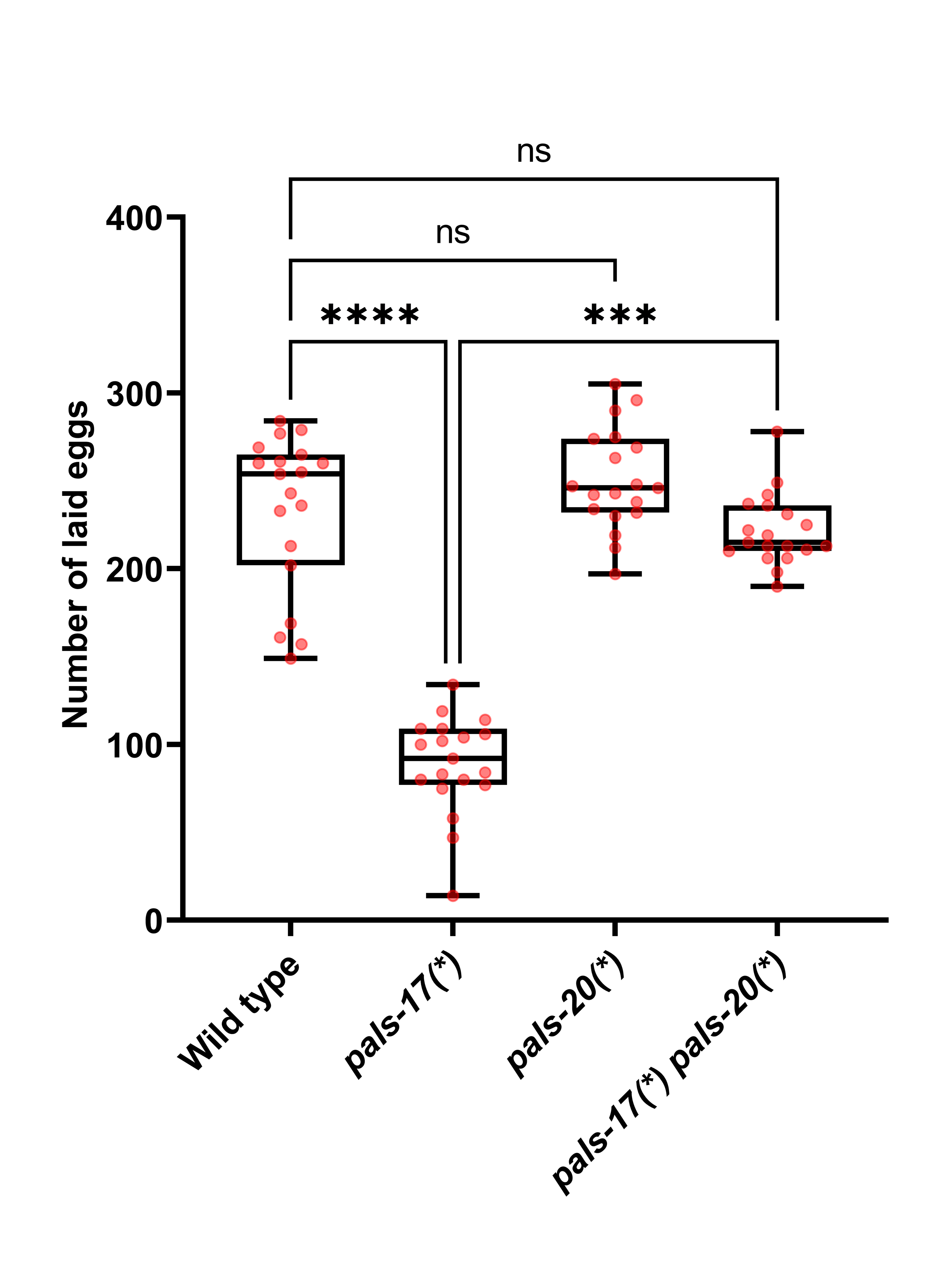

Supplement: S5 Fig — elegans. pals-17 mutants have significantly lower brood sizes, and this phenotype is pals-20-dependent. Brood size measurements are shown as a box-and-whisker plot for wild-type, pals-17(*), pals-20(*) and pals-17(*) pals-20(*) animals. Box lines represent the median values, box bounds indicate 25th and 75th percentiles, and whiskers extend to the minimum and maximum values. Red dots represent individual values for each animal; 19 animals were analyzed for each strain (at least five animals per each of the three experimental replicates). A Kruskal-Wallis test was used to calculate p-values; **** p < 0.0001; *** p < 0.001; ns indicates no significant difference. (TIF) [file ppat.1011120.s005.tif]

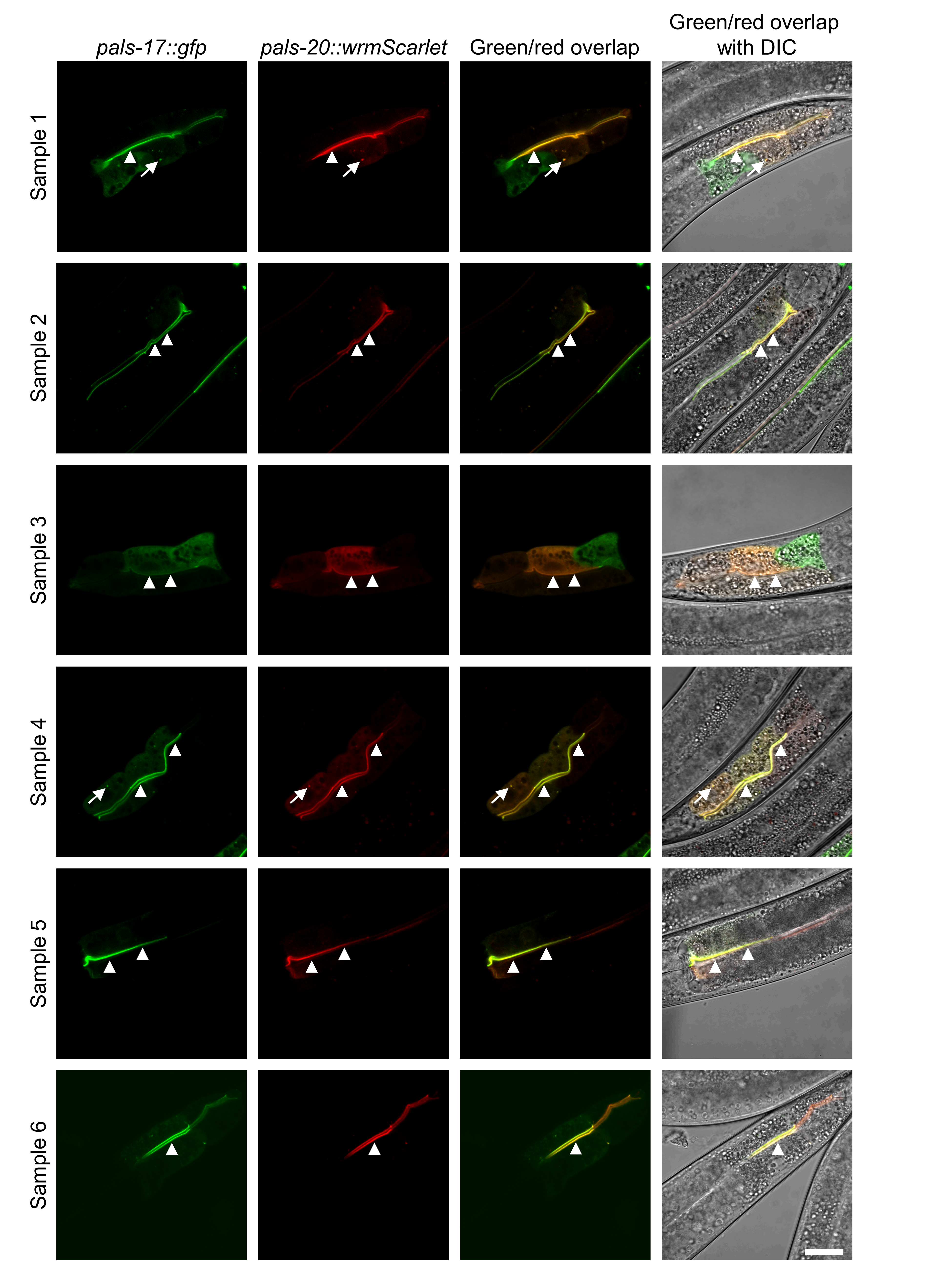

Supplement: S6 Fig — Expression of PALS-17::GFP and PALS-20::wrmScarlet translational reporters and their colocalization. Representative images showing separate green and red channels, as well as their overlap and overlap with DIC. Arrowheads indicate colocalization of fluorophores at the border with the intestinal lumen; arrows indicate areas of colocalization in other regions of intestinal cells. Scale bar, 20 μm. (TIF) [file ppat.1011120.s006.tif]

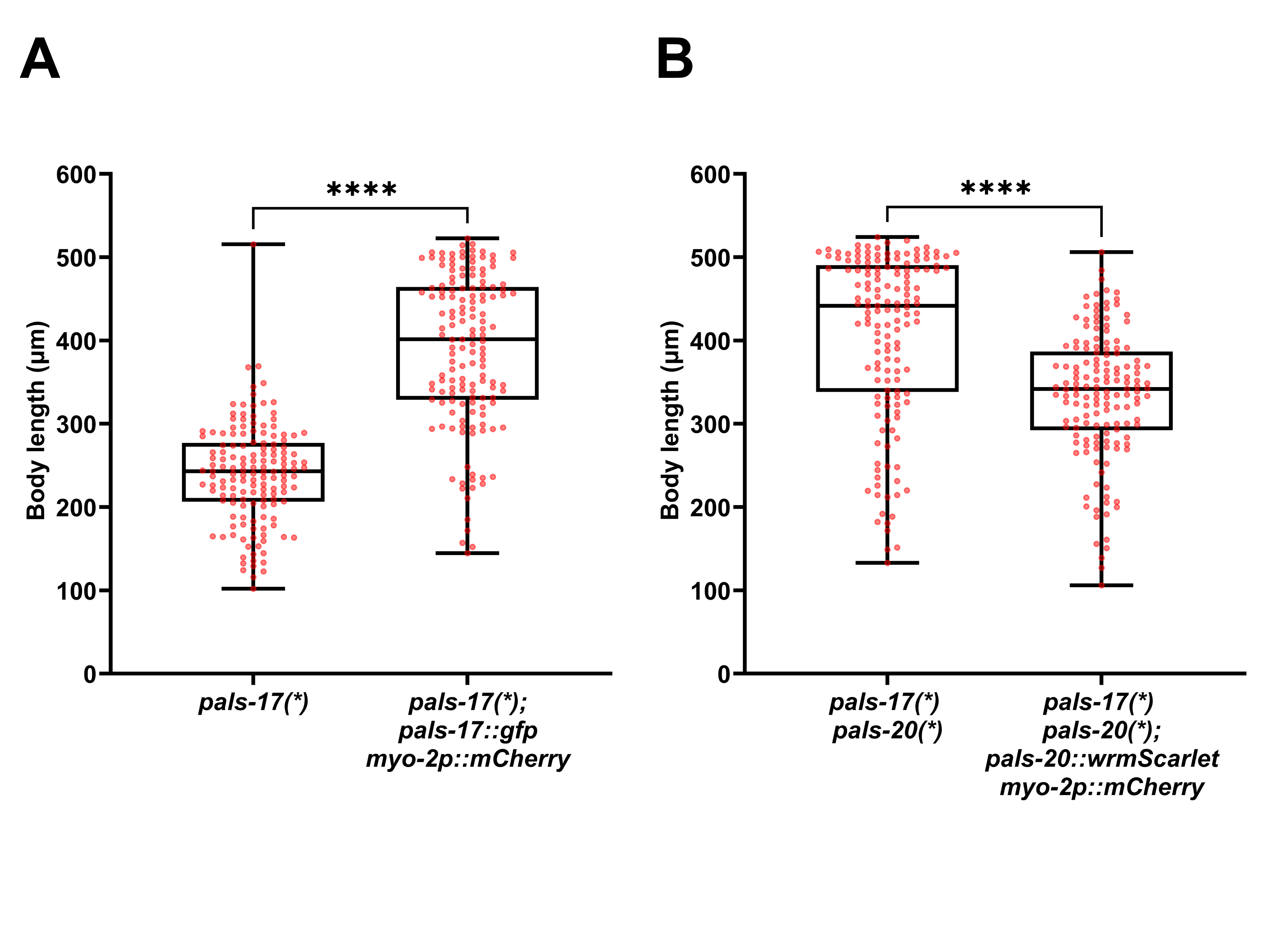

Supplement: S7 Fig — (A, B) Body length measurements are shown as box-and-whisker plots for pals-17(*) (A) and pals-17(*) pals-20(*) animals (B). Animals expressing pals-17::gfp myo-2p::mCherry (A) and pals-20::wrmScarlet myo-2p::mCherry arrays (B) as well as their non-transgenic siblings were analyzed. Box lines represent median values, box bounds indicate 25th and 75th percentiles, and whiskers extend to the minimum and maximum values. Red dots represent individual values for each animal; 50 animals per each of the three experimental replicates were analyzed. A Kolmogorov-Smirnov test was used to calculate p-values; **** p < 0.0001. (TIF) [file ppat.1011120.s007.tif]

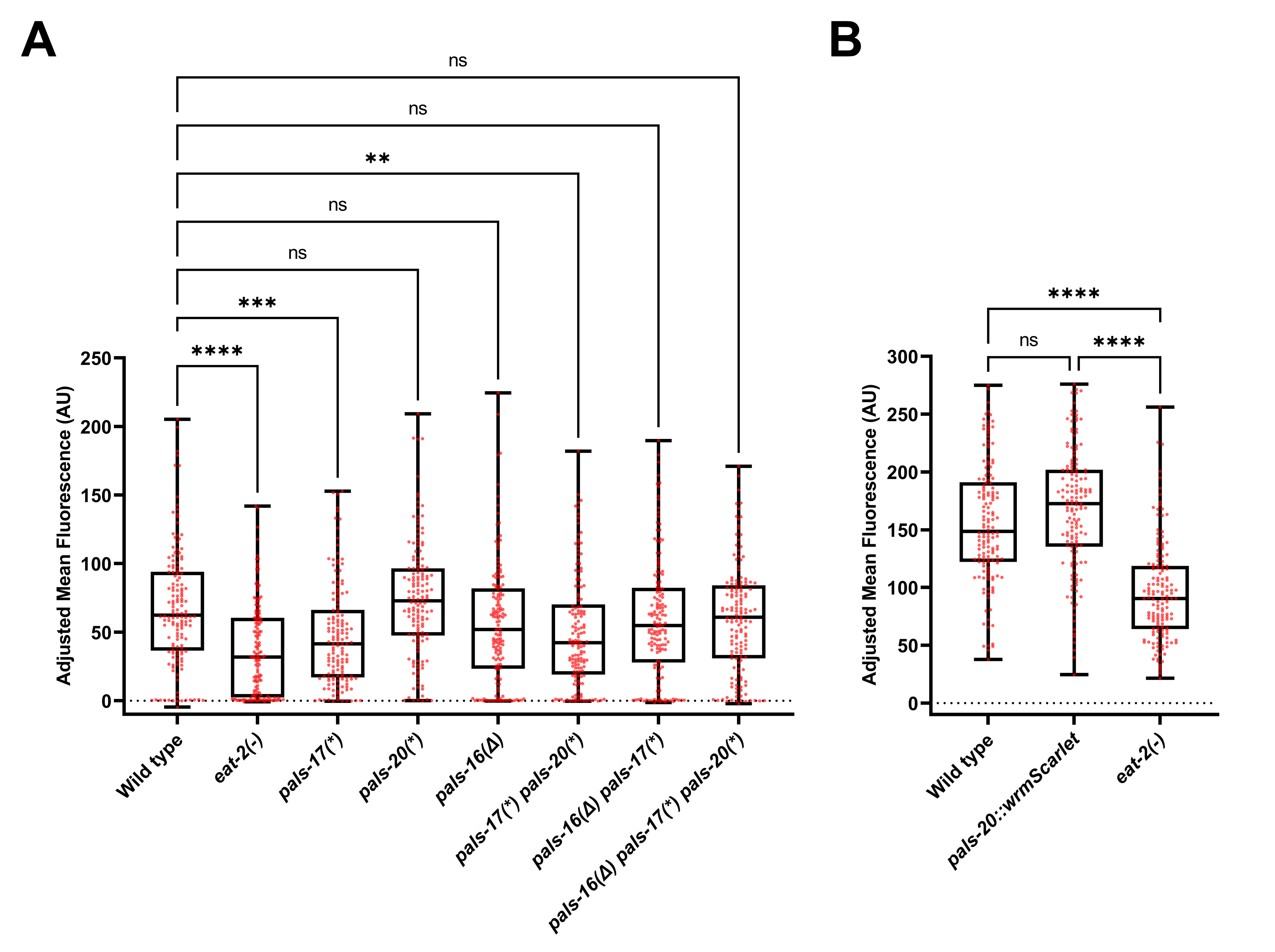

Supplement: S8 Fig — (A, B) Box-and-whisker plots of bead fluorescence levels per animal. Box lines represent median values, box bounds indicate 25th and 75th percentiles, and whiskers extend to the minimum and maximum values. Data from three independent experimental replicates are shown. Red dots represent individual values for each animal; 50 animals were analyzed per strain per replicate. eat-2(ad465) mutant was used as a feeding-defective control. A Kruskal-Wallis test was used to calculate p-values; **** p < 0.0001; *** p < 0.001; ** p < 0.01; ns indicates no significant difference. AU = arbitrary units. (TIF) [file ppat.1011120.s008.tif]

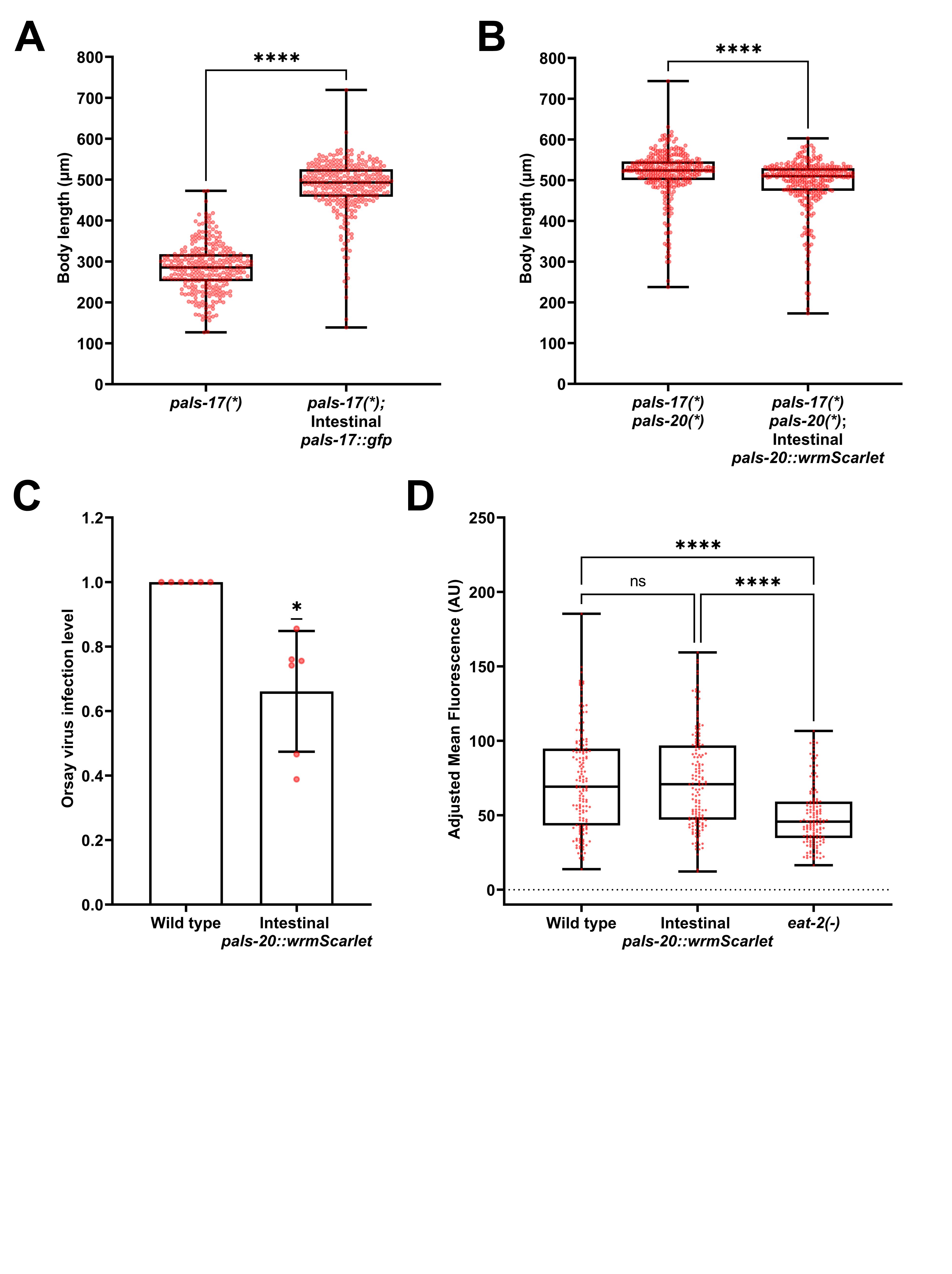

Supplement: S9 Fig — (A, B) Body length measurements are shown as box-and-whisker plots for pals-17(*) (A) and pals-17(*) pals-20(*) animals (B). Animals carrying intestinal pals-17::gfp (A) and intestinal pals-20::wrmScarlet arrays (B) as well as their non-transgenic siblings were analyzed. Box lines represent median values, box bounds indicate 25th and 75th percentiles, and whiskers extend to the minimum and maximum values. Red dots represent individual values for each animal; 100 animals per each of the three experimental replicates were analyzed. A Kolmogorov-Smirnov test was used to calculate p-values; **** p < 0.0001. (C) Infection levels of animals overexpressing pals-20::wrmScarlet construct in the intestine and their non-transgenic siblings (wild type) that were infected with Orsay virus. Animals were scored based on the presence or absence of the Orsay virus-specific FISH probe fluorescence (six experimental replicates, at least 100 animals per replicate). Red dots represent the infection level for each replicate; the wild type was set at one for each replicate. Error bars are SD. A one sample Wilcoxon test was used to calculate the p-value; * p < 0.05. (D) Box-and-whisker plots of bead fluorescence levels per animal. Box lines represent median values, box bounds indicate 25th and 75th percentiles, and whiskers extend to the minimum and maximum values. Data from three independent experimental replicates are shown. Red dots represent individual values for each animal; 50 animals were analyzed per strain per replicate. eat-2(ad465) mutant was used as a feeding-defective control. A Kruskal-Wallis test was used to calculate p-values; **** p < 0.0001; ns indicates no significant difference. AU = arbitrary units. (TIF) [file ppat.1011120.s009.tif]
